# *Gleditsia sinensis* Thorn Attenuates the Collagen-Based Migration of PC3 Prostate Cancer Cells through the Suppression of α2β1 Integrin Expression

**DOI:** 10.3390/ijms17030328

**Published:** 2016-03-02

**Authors:** Sujin Ryu, Ki Moon Park, Seung Ho Lee

**Affiliations:** 1 Department of Nano-Bioengineering, Incheon National University, 119 Academy-ro, Yeonsu-gu, Incheon 406-772, Korea; lovedbtnwls@naver.com; 2 Department of Food Science and Biotechnology, Sungkyunkwan University, Seoul 440-746, Korea; pkm1001@skku.edu

**Keywords:** *Gleditsia sinensis*, prostate cancer, α2β1 integrin, collagen, migration

## Abstract

*Gleditsia sinensis* thorns (GST) have been used as a traditional medicine for carbuncles and skin diseases. The purpose of this study was to decide whether non-toxicological levels of water extract of GST (WEGST) are effective in inhibiting the progress of prostate cancer formation and to identify the target molecule involved in the WEGST-mediated inhibitory process of prostate cancer cell migration and *in vivo* tumor formation. Through the Boyden chamber migration assay, we found that non-toxic levels of WEGST could not attenuate the PC3 migration to the bottom area coated with serum but significantly inhibited PC3 cell migration to the collagen-coated bottom area. We also found that non-toxic levels of WEGST significantly attenuated collagen against adhesion. Interestingly, ectopic administration of WEGST could not affect the expression of α2β1 integrin, which is known as a receptor of collagen. However, when the PC3 cells adhered to a collagen-coated plate, the expression of α2 integrin but not that of β1 integrin was significantly inhibited by the administration of non-toxic levels of WEGST, leading to the inhibition of focal adhesion kinase (FAK) phosphorylation. Furthermore, oral administration of WEGST (25 mg/kg/day) significantly inhibited the size of a PC3 cell-xenografted tumor. Taken together, these results suggest a novel molecular mechanism for WEGST to inhibit prostate cancer progression at particular stages, such as collagen-mediated adhesion and migration, and it might provide further development for the therapeutic use of WEGST in the treatment of prostate cancer progression.

## 1. Introduction

Prostate cancer is a common cancer occurring in male individuals and the second leading cause of cancer-related death in men [1]. Several treatments, such as androgen deprivation, prostatectomy, and radiation therapy have been performed to cure patients suffering from prostate cancer. However, a high rate of mortality has been detected, mainly due to the metastasis of prostate cancer cells that these therapies have failed to remove [2]. Metastasis is initiated when an extremely small population of cancer cells invades from primary tumor to surrounding tissues and then intravasates into blood vessels. During metastasis, cancer cells migrate to different areas and adapt to the new environment by interacting with the extracellular matrix (ECM). Although many anti-cancer agents focus on attenuating the proliferation of cancer cells, the development of therapeutics that target the metastatic progress, such as migration and adhesion to the ECM, may provide alternative strategies for the treatment of prostate cancer.

Integrin is a well-known transmembrane protein that has a central role in metastasis [3]. It is composed of two distinct chains of α and β subunits [4,5], and the 18 α and eight β subunits construct 24 unique heterodimeric integrins responsible for cellular interaction with the ECM, such as collagen, fibronectin, and laminin. Each integrin can bind a specific ligand, and the interaction between integrin and the ECM induces the cluster formation of integrin with downstream signaling molecules, such as focal adhesion kinase (FAK) molecules, resulting in lamellipodium formation and cell migration. Since each cancer shows different expression pattern of integrins, targeted inactivation of specific integrin of cancer cells may be an effective way to regulate the metastatic properties of cancer cells. In the case of prostate cancer, α2β1 integrin, which is a receptor for collagen [6], seems to be dominantly expressed and to have more critical role in the invasion of PC3 cells than other kinds of integrin [7]. Therefore, the development of therapeutics that can attenuate the function of α2β1 integrin may be effective in inhibiting the metastasis of prostate cancer.

*Gleditsia sinensis* (GS) has been utilized as Oriental medicine to cure diverse diseases, such as carbuncles and swelling [8]. Biological properties of different parts of GS (fruits, seed, radix, and radix cortex) have been suggested for treating several diseases, such as suppuration, carbuncles, and obesity [9,10,11]. Moreover, ethanol extract of *Gleditsia sinensis* thorns (GST) seems to have anti-angiogenic effects [12,13]. In addition, the anti-tumor activity of GST has been demonstrated against gastric cancer, colon cancer, and lung cancer [13,14,15,16]. However, these studies focused on the suppressive effect of GST in cell cycle progression and cell proliferation. In our study, we evaluated the regulatory effects and molecular mechanisms of GST, particularly in the migration and adhesion of prostate cancer cells. Moreover, we examined the *in vivo* efficacy of GST by using a tumor-bearing nude mouse model.

## 2. Results

### 2.1. Non-Toxic Levels of Water Extract of GST (WEGST) Are Effectively Attenuated by the Collagen-Mediated Migration of PC3 Prostate Cancer Cells

**Figure 1:**
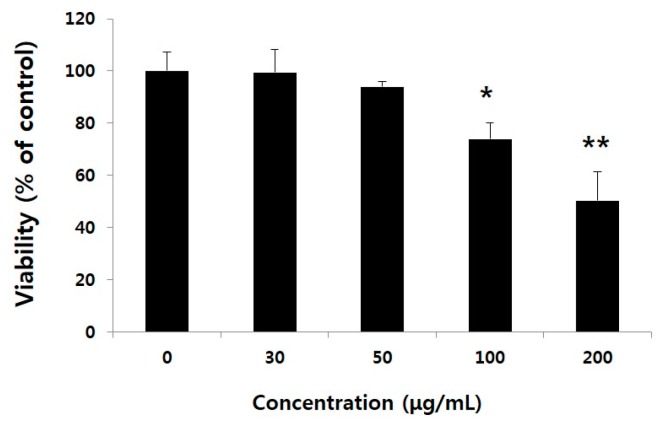
Effect of water extract of *Gleditsia sinensis* thorns (WEGST) on PC3 cell viability. PC3 cells were treated with various concentrations of WEGST (0–200 µg/mL) for 24 h. Cell viability was measured using the WST-1 assay kit. Each value is expressed as the mean ± standard deviation (SD) of three wells. Asterisks (*) refers to statistical differences compared to the control (non-treated). * *p* < 0.05 and ** *p* < 0.001.

We first examined the cytotoxicity of water extract of GST (WEGST) on PC3 cells; the administration of WEGST up to a concentration of 50 µg/mL did not show cytotoxicity in the cells. The viability of the PC3 cells, however, decreased significantly compared to a concentration of 100 µg/mL of WEGST (Figure 1). Based on these findings, to determine the effect of non-toxic levels of WEGST on PC3 cells, we next analyzed the effect of WEGST (50 µg/mL) on PC3 cell migration. Since the process of adhesion and migration of cancer cells is controlled by interactions with the ECM, using the Boyden chamber assay, we assessed the effect of non-toxic levels of WEGST on PC3 cell migration against serum and collagen.

**Figure 2:**
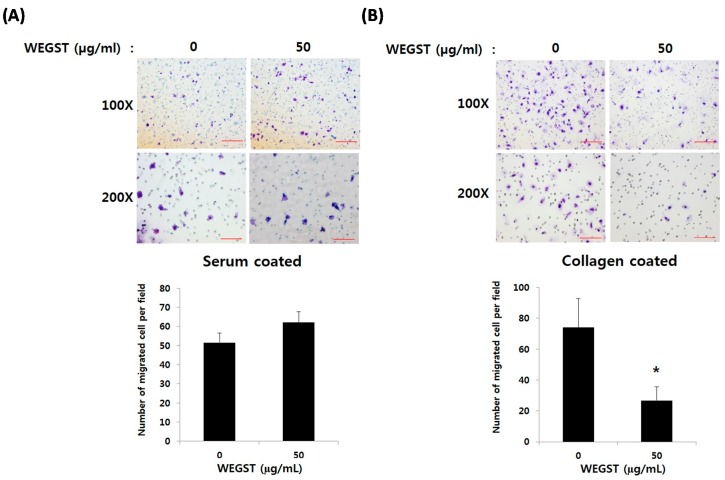
WEGST-attenuated collagen-mediated migration. PC3 cells were pre-treated with WEGST (50 µg/mL) for 6 h and seeded in a chamber of 20% serum (**A**) and collagen I (**B**) coated on the bottom layer of the Transwell membrane. Cells that migrated to the bottom part of the Transwell membrane were fixed with paraformaldehyde (4%, *w*/*v*) for 10 min and then stained with crystal violet solution (0.5%, *w*/*v*), and counted. A representative figure of three independent experiments is shown in the upper panels, with two different magnifications (100× and 200×). The number of cells was counted in four different fields and tabulated in the bottom panel. Error bars refer to the SD of the mean. Bar = 0.1 µm of 200× magnification and 0.2 µm of 100× magnification. * *p* < 0.05, compared with non-treated PC3 cells (0 µg/mL of WEGST).

Figure 2A shows that non-toxic levels of WEGST (50 µg/mL) did not affect the migration of PC3 cells from the upper side of the membrane to the bottom area of the membrane, which was coated with serum. However, the administration of WEGST (50 µg/mL) to the PC3 cells significantly attenuated the migration to the bottom area of the membrane, which was coated with collagen (Figure 2B). These data suggest that the administration of non-toxic levels of WEGST could be an effective way to inhibit prostate tumorigenesis at specific stages, such as prostate cancer cell migration toward the collagen.

### 2.2. Collagen-Mediated Expression of α2 Integrin in PC3 Prostate Cancer Cells Is Attenuated by the Administration of WEGST

**Figure 3:**
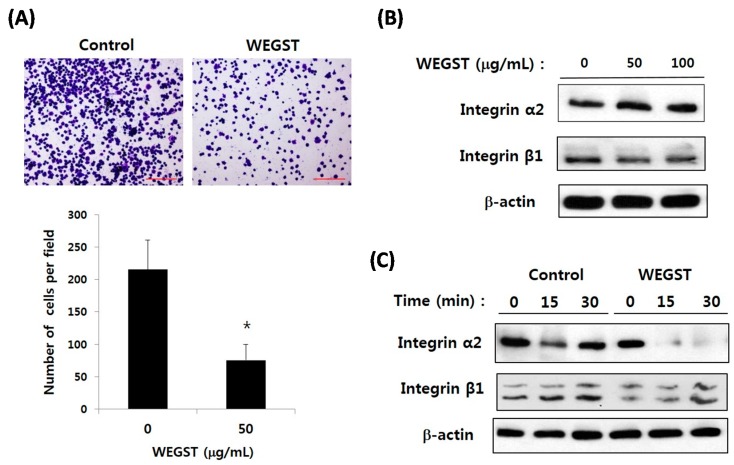
WEGST treatment attenuated the cell adhesion and the expression of α2 integrin; the inhibitory effect of WEGST on PC3 cell migration was investigated. A representative figure of three independent experiments is exhibited in the upper panels. The number of attached cells was counted in four different fields and tabulated in the bottom panel (**A**); Semi-confluent PC3 cells were treated with WEGST; and the expression of α2 and β1 integrin was examined by western blotting (**B**); PC3 cells were pre-treated with WEGST and seeded on a collagen-coated plate. PC3 cells were harvested at various times, as indicated, and the expression of α2 and β1 integrin was examined by western blotting (**C**). The results shown are representative of at least three independent experiments. Error bars refer to the SD of the mean. Bar = 0.2 µm. * *p* < 0.05, compared with control (non-treated).

Since non-toxic levels of WEGST were proved to inhibit the PC3 cell migration only toward collagen (Figure 2), we next examined the effect of WEGST on direct interactions between PC3 cells and collagen through adhesion assay. Figure 3A shows that non-toxic levels of WEGST markedly inhibited the PC3 cell adhesion on collagen. Based on these data, we then investigated the target molecules that were responsible for the inhibitory effect of WEGST in collagen-mediated adhesion and migration of PC3 cells; α2β1 integrin is a well-known receptor for collagen, and it has been reported as a dominant cell-surface protein among several integrin family members that promote the cell adhesion and migration of PC3 cells [17]. We therefore wanted to examine the effect of WEGST on the expression of α2β1 integrin in PC3 cells. Figure 3B shows that the expression levels of α2β1 integrin in PC3 cells were not affected by the administration of WEGST up to a concentration of 100 µg/mL. Interestingly, however, the expression of α2 integrin (but not of β1 integrin) was gradually inhibited by pre-treatment with WEGST (50 µg/mL) during the process of adhesion against collagen (Figure 3B). These results suggest that non-toxic levels of WEGST could be effective in inhibiting the interactions between PC3 cells and collagen by attenuating the expression of α2 integrin.

### 2.3. Collagen-Mediated Intracellular Focal Adhesion Kinase (FAK) Signaling of PC3 Prostate Cancer Cells Is Inhibited by Administration of WEGST

**Figure 4:**
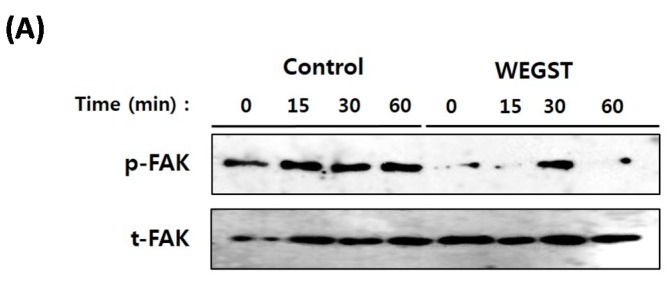

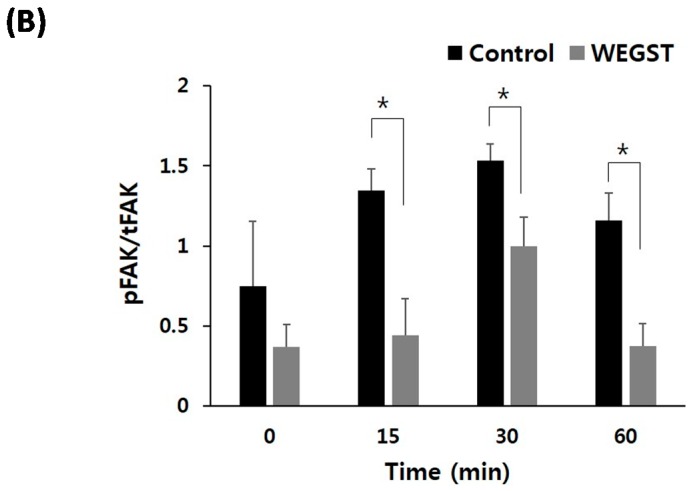
Collagen mediated activation of focal adhesion kinase (FAK) was impaired by WEGST treatments. The quiescent cells were pre-incubated with or without WEGST (50 µg/mL) and seeded in the collagen-coated plated, and then harvested at the indicated times. Cell lysates were western blotted with anti-phosphor FAK antibody, and the membrane was reproved and reacted with anti-FAK antibody for the detection of FAK expression. A representative figure of three independent experiments is shown (**A**); the activation ratio of phosphor FAK (p-FAK)/total FAK (t-FAK) is tabulated in the bottom panel (**B**). * *p* < 0.05, compared with control.

To further elucidate the molecular mechanisms by which WEGST regulates adhesion and migration against collagen, we investigated the change in activation of FAK, which plays a pivotal role in integrin-mediated adhesion and migration. As expected, the activation/phosphorylation of FAK was increased when the normal PC3 cells were attached to the collagen-coated plate, but the PC3 cells treated with non-toxic levels of WEGST exhibited attenuated levels of phosphorylation of FAK (Figure 4). These data suggest that WEGST could effectively attenuate integrin-mediated signaling when the PC3 cells interacted with collagen during the adhesion process.

### 2.4. Oral Administration of WEGST Inhibits in Vivo Tumor Formation

**Figure 5:**
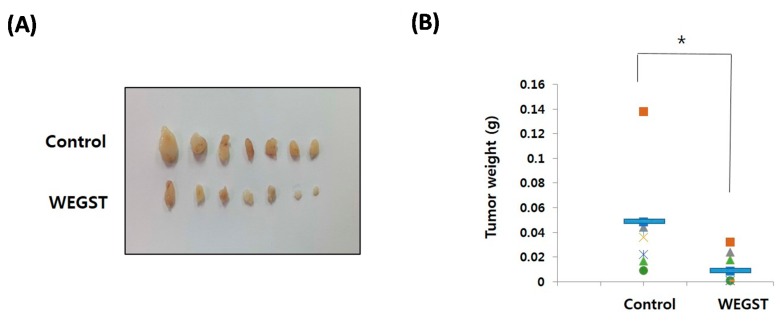

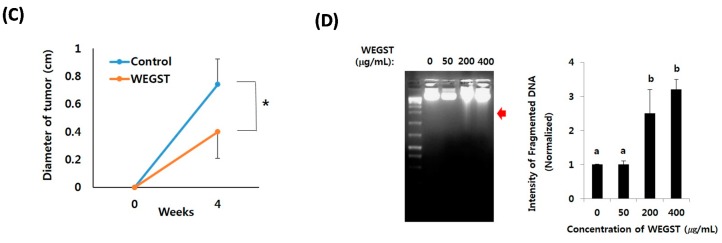
Oral administration of WEGST attenuated tumor formation. PC3 cells were injected subcutaneously. From the day after the inoculation of the PC3 cells, WEGST (25 mg/kg/day) was administered orally to the WEGST mouse group (*n* = 7) every day. Four weeks later, all mice were sacrificed, and then the tumors were removed and weighed. Tumors of each group are shown (**A**); and the wet weights (**B**) and sizes (**C**) of seven tumors are illustrated; the weights and sizes of the tumors in the WEGST-administered mouse group were significantly decreased compared with those of the control mouse group. PC3 cells were incubated with various concentrations (0–400 µg/mL) of WEGST, and the fragmented DNA was extracted and resolved in 1.2% agarose gel. Representative figure of three independent experiments are shown (**left** panel). The intensities of fragmented DNA areas of three independent experiments were measured using Image J software (Softonic international, SF, USA) and then tabulated (**right** panel; normalized by intensity of non-treated cells; 0 µg/mL of WEGST) (**D**). The red arrow indicates fragmented DNA, while the different letters (a, b) in (**D**) indicate significant differences (*p* < 0.05) between groups, * *p* < 0.05 compared with control mouse groups, using the Mann-Whitney *U* test.

To further verify the anti-tumor effects of WEGST, oral administration of WEGST (25 mg/kg/day) was initiated from day 2 after the inoculation of PC3 cells into the subcutaneous areas of nude mice. As shown in Figure 5, the weight and size of the tumors in the WEGST-administered mouse group was significantly decreased compared with the control mouse group. However, there were no significant differences in body weights between control and WEGST mice at the end of the experiment (control: 33.2 ± 2.1 g, WEGST: 34.08 ± 1.7 g). Finally, to support the anti-tumor effect of WEGST *in vivo*, we investigated whether WEGST has the ability to induce programed cell death in PC3 cells. Interestingly, administration of low concentration of WEGST (50 µg/mL) did not induce DNA fragmentation, but fragmented DNA was detected in PC3 cells treated with high concentrations of WEGST (from a concentration of 200 µg /mL; Figure 5D). Taken together, these data indicate that oral administration of WEGST is effective for inhibiting *in vivo* tumor formation partly by inducing the PC3 cell death.

## 3. Discussion

GST has long been used as an Oriental medicine, and it has been shown to have anti-allergenic, anti-microbial, and anti-mutagenic effects. Several bioactive compounds with anti-microbial and anti-mutagenic components have been suggested; lupane acid, isolated from GS, was reported as a bioactive component that has strong anti-human immunodeficiency virus (HIV) activity [18]. In addition, several phenolic compounds, including ethyl gallate and eriodictyol, were also reported as active compounds of GS that exhibit anti-bacterial activity [19]. Lim *et al.* [20] isolated one triterpenoid and four steroids from GST, and stigmasterol was identified as the bioactive compound with the highest anti-mutagenic activity among them. Although these researchers employed an *in vitro* assay system using a bacterial strain, stigmasterol did not inhibit the growth of bacteria; rather, it attenuated the mutagen-induced decrease of bacterial β-galactosidase activity. Interestingly, lupane-type triterpenoids, isolated from other herbs, showed anti-tumor effects [21,22], and ethyl gallate was reported to have an anti-invasive effect related to breast cancer [23]. Stigmasterol, isolated from *Azadirachta indica*, was also reported to have a chemo-preventive effect on skin cancer [24]. Taken together, there is a possibility that lupine acid, ethyl gallate, and stigmasterol are active compounds that can account for the anti-tumor effects of GS.

GST has been reported to have anti-proliferative effects on gastric cancer [25] and colon cancer [14,15] by attenuating the cell cycle progression. However, the molecular mechanism that underlies the effect of GST in cancer cell migration and adhesion has yet to be elucidated. In the present study, we focused on determining whether non-toxic levels of WEGST have anti-cancer effects in PC3 prostate cancer cells. Since many therapeutic candidates have been tested to determine whether they exert enough activity to inhibit proliferation accompanied by cell death, relatively high doses of agents have been used to show the anti-cancer effects of WEGST. Considering the high levels of side effects that are experienced by cancer patients undergoing chemotherapy, however, it is crucial to determine the effective range with the lowest dosage of anti-cancer therapeutics.

In our study, it was interesting to note that the administration of non-toxic levels of WEGST significantly inhibited the collagen-mediated migration and adhesion of prostate cancer PC3 cells. WEGST’s anti-tumor activities were found to be mediated through the attenuation of collagen-mediated expression of α2 integrin and its intracellular signaling (phosphorylation of FAK). These findings suggest that WEGST effectively inhibits the interaction between collagen in the ECM area and α2β1 integrin in PC3 prostate cancer cells.

α2β1 Integrin is a well-known cell-surface protein that is responsible for cell adhesion to ECM; both the expression, and the activation/phosphorylation of α2β1 integrin has been implicated in prostate cancer progression. It was reported that PC3 cells migrated most vigorously toward collagen than any other ECM proteins, and α2β1 integrin seems to have important role in the invasion of PC3 cells [7]. Thus, we specifically investigated the effect of WEGST on PC3 cell migration and adhesion with respect to collagen. Since cell adhesion and migration are important steps in metastasis, the targeted inactivation of α2β1 integrin could be a promising strategy for treating the progression of prostate cancer. Since our data showed that WEGST has anti-tumor activities through the attenuation of collagen-mediated expression of α2 integrin, there is a possibility that WEGST may effectively attenuate the migration and adhesion of other tumor lines that express α2β1 integrin.

The most common cause of death among prostate cancer patients is metastasis, and many prostate cancer patients suffer from the side effects of chemotherapy drugs; as such, even if the use of low doses of therapeutics may not be effective for inhibiting the proliferation of prostate cancer cells, if such treatment could be effective for inhibiting metastasis, it could be an attractive way to treat prostate cancer patients. Since our data suggested that administration of non-toxic levels of WEGST showed inhibitory effects in collagen against migration and adhesion of PC3 cells, WEGST may more effectively attenuate the progression of late stage (metastatic) tumors than that of early stage (proliferative) tumors.

Taken together, to the best of our knowledge, this present study provides the first evidence that WEGST inhibits the collagen-mediated adhesion and migration of prostate cancer PC3 cells via the inactivation of α2β1 integrin. Furthermore, the oral administration of WEGST to PC3-cell-derived tumor-bearing nude mouse groups reduced the size of tumors compared with non-treated tumor-bearing nude mouse groups. Although we did not demonstrate the detailed physiological or molecular parameters concerning how WEGST attenuated the *in vivo* tumor formation, our data provide the first *in vivo* data on the efficacy of WEGST in the presence of prostate cancer. Our future research will involve a molecular-based study to determine whether WEGST attenuates metastasis from the prostate to the lymph node using an orthotopic prostate tumor model. In conclusion, these findings provide momentous insights into the pharmacological efficacy of GST in prostate cancer progression, and support GST’s future development as a potent anti-cancer agent for the treatment of prostate cancer.

## 4. Materials and Methods

### 4.1. Preparation of WEGST

One hundred grams of GST were extracted with 1 L of hot water. The supernatant was filtered, concentrated with rotary evaporation (Heidolph Instruments GmbH & Co., Schwabach, Germany), and lyophilized. The yield of the dried extract was approximately 6.8 g/L.

### 4.2. Cell Culture and Cytotoxicity Assay

PC3 prostate cancer cells were obtained from the American Type Culture Collection (ATCC; Manassas, VA, USA) and maintained in Roswell Park Memorial Institute (RPMI) 1640, supplemented with 10% fetal bovine serum (FBS; HyClone Laboratories, Logan, UT, USA), penicillin (100 U/mL), and streptomycin (100 µg/mL). The PC3 cells were seeded in a 96-well plate (5 × 10^3^ cells/well) and cultured for 24 h. WEGST was added to the culture media at various concentrations (30–200 µg/mL), and it was incubated in a CO_2_ incubator during 24 h. WST-1 solution (Corning, Corning, NY, USA) was then added and incubated for 2 h. The absorbance was calculated at 450 nm with a microplate reader (Bio-Rad, Hercules, CA, USA).

### 4.3. Collagen against Migration Assay

Cell migration was assayed using an 8 µm pore size Transwell Permeable Support (BD Biosciences, San Jose, CA, USA). The bottom part of the Transwell membrane was coated with collagen I (10 µg/mL, Sigma, St. Louis, MO, USA) or serum (20% *w*/*v*) in phosphate-buffered saline (PBS) at 4 °C overnight. The PC3 cells were pre-incubated with WEGST (50 µg/mL) for 6 h, and then 5 × 10^4^ cells were added to the upper chamber. After further incubation for 2 h (collagen-coated Transwell) or 6 h (serum-coated Transwell) at 37 °C in a CO_2_ incubator, any cells that reached the bottom layer were fixed with paraformaldehyde (4%, *w*/*v*) and then stained with crystal violet (0.5%, *w*/*v*) and counted under a microscope.

### 4.4. Cell Adhesion Assay

The PC3 cells were harvested and pre-incubated with and without WEGST (50 µg/mL); 2 × 10^5^ cells were then seeded on collagen I (10 µg/mL)-coated 96-well plates. After further incubation for 15 min at room temperature, the plates were washed, fixed, and stained with 5% crystal violet solution. The number of attached cells was then counted under a microscope.

### 4.5. Analysis of Collagen against Expression of α2β1 Integrin

To determine the effect of collagen against the expression of α2β1 integrin, PC3 cells were harvested and pre-treated with and without WEGST (50 µg/mL) for 30 min; 3 × 10^5^ cells were then seeded on collagen I (10 µg/mL)-coated plates and harvested at various times, as indicated. The cells were solubilized in lysis buffer composed of 20 mM Tris-HCl (pH 7.4), 5 mM sodium pyrophosphate, 5 mM EDTA, 150 mM NaCl, Nonidet P-40 (1%, *w*/*v*), 10 mM NaF, 1 mM sodium orthovanadate, 1 mM phenylmethylsulfonyl fluoride, 10 mM β-glycerophosphate, and a protease inhibitor mixture (Sigma-Aldrich Co., St. Louis, MO, USA). Equal amounts of cell lysates were separated by sodium dodecyl sulfate polyacrylamide gel electrophoresis (SDS-PAGE) and transferred to polyvinylidene difluoride (PVDF) membranes. The membranes were incubated separately with monoclonal anti-β1 integrin antibody (sc-374429; Santa Cruz, CA, USA), rabbit anti-α2 integrin antibody (Merck Millipore, Gibbstown, NJ, USA), and mouse monoclonal anti-β-actin antibody (Santa Cruz, CA, USA), followed by horseradish peroxidase (HRP)-conjugated anti-rabbit immunoglobulin G (IgG); enhanced chemiluminescence (ECL) reagents (Bio-Rad Co., Hercules, CA, USA) were used to detect signals.

### 4.6. Analysis of FAK Phosphorylation

Semi-confluent PC3 cells were rinsed twice with PBS and separated into single cells by treatment with trypsin/EDTA solution (HyClone Laboratories, Logan, UT, USA). Trypsin inhibitor from glycine max soybean (Sigma-Aldrich Co.) was added to deactivate the trypsin. The cells were pre-incubated with and without WEGST (50 µg/mL) for 30 min; 3 × 10^5^ cells were then seeded on collagen I (10 µg/mL)-coated plates and harvested at various times, as indicated. Total protein was extracted from each cell and transferred to PVDF membranes, as described above. The membranes were first used for the detection of phosphor-FAK expression using the phosphospecific antibody (p-FAK; BD Biosciences, San Jose, CA, USA), and the blot was stripped through incubation with 1 N NaOH during 1–2 min at room temperature; it was then washed three times with TBS-T and used for the detection of total FAK (t-FAK) using the mouse anti-FAK antibody (BD Biosciences, San Jose, CA, USA). The activation rate of FAK (p-FAK/t-FAK) was estimated using the Image J program.

### 4.7. DNA Fragmentation Assay

Semi-confluent PC3 cells were rinsed twice with PBS and incubated with various concentrations (0–400 µg/mL) of WEGST at 37 °C in a CO_2_ incubator for 48 h. Total DNA was extracted using the AccuPrep genomic DNA extraction kit (Bioneer Co., Seoul, Korea) according to the manufacturer’s protocol. Extracted DNA was analyzed via agarose gel (1.2%, *w*/*v*) electrophoresis. DNA was stained with ethidium bromide (EtBr) and visualized using a NaBI gel documentation system (KoreaLabTech Co., Seoul, Korea).

### 4.8. Xenograft Tumor Assay

BALBc nude (nude/nude) mice (6- to 8-week-old males) obtained from NARA Biotech (Seoul, Korea) were used for the subcutaneous tumor cell injection. The mice were housed in stainless steel cages under controlled temperature (23 ± 3 °C), humidity (55% ± 10%), and photoperiod (12 h cycles of light and dark). All of the animals were fed standard mouse food (Orient Bio, Seoul, Korea) and water *ad libitum*. For the purposes of acclimation, all of the mice were kept in cages for 1 week before commencing the experiment. All experiments were performed in accordance with the standards of the Animal Research Committee of Sungkyunkwan University (SKKUIA CUC-20150037). The mice (*n* = 14) were divided into two groups (control and WEGST) and anesthetized with Avertin. Following this, 2 × 10^6^ PC3 cells were suspended in 150 µL of RPMI 1640 medium and inoculated into the subcutaneous area. Starting the day after the inoculation of the PC3 cells, WEGST (25 mg/kg/day) was orally administered to the WEGST mouse group (*n* = 7) every day. Four weeks later, the mice were sacrificed, and then the tumors were removed and weighed. The specimens were preserved by fixation in neutral buffered formalin.

### 4.9. Statistical Analysis

The data are presented as mean ± SD; one-way analysis of variance (ANOVA) in the XLSTAT program (Addinsoft, New York, NY, USA) was used to analyze the data. A Student-Newman-Keuls test was then implemented; a *p*-value of <0.05 was considered to be significant.

## 5. Conclusions

In our study, we found that WEGST attenuates the collagen-mediated adhesion and migration of prostate cancer PC3 cells through the inactivation of α2β1 integrin. Furthermore, we also found the *in vivo* efficacy of WEGST to reduce the size of the PC3-cell-derived tumor. Collectively, our data provide pharmacological roles of WEGST in regulating prostate cancer PC3 cells and suggest further development of WEGST as a therapeutic agent for preventing and treatment of prostate cancer.
